# Multivariate Analysis Compares and Evaluates Heat Tolerance of Potato Germplasm

**DOI:** 10.3390/plants13010142

**Published:** 2024-01-04

**Authors:** Sujie Zhang, Han Ye, Lingshuang Kong, Xiaoyu Li, Yeqing Chen, Shipeng Wang, Bailin Liu

**Affiliations:** 1 State Key Laboratory for Crop Stress Resistance and High-Efficiency Production, College of Agronomy, Northwest A&F University, Yangling 712100, China; 2 Shenzhen Research Institute, Northwest A&F University, Shenzhen 518000, China

**Keywords:** cultivars, heat stress, multivariate analysis, cultivar screening

## Abstract

High temperature is the most important environmental factor limiting potato (*Solanum tuberosum* L.) yield. The tuber yield has been used to evaluate the heat tolerance of some potato cultivars, but potato yield was closely correlated with the maturation period. Therefore, it is necessary to employ different parameters to comprehensively analyze and evaluate potato tolerance to heat stress. This study aimed to investigate physiologic changes during growth and development, and develop accurate heat tolerance evaluation methods of potato cultivars under heat stress. About 93 cultivars (including foreign elite lines, local landraces and cultivars) were screened using an in vitro tuber-inducing system (continuous darkness and 8% sucrose in the culture medium) under heat stress (30 °C) and normal (22 °C) conditions for 30 days. The tuber yield and number decreased significantly under heat stress compared to the control. A total of 42 cultivars were initially selected depending on tuber formation, after in vitro screening, further testing of selected cultivars was conducted in ex vitro conditions. The screened cultivars were further exposed to heat stress (35 °C/28 °C, day/night) for 60 days. Heat stress led to an increase in the plant height growth rate, fourth internode growth rate, and membrane damage, and due to heat-induced damage to chloroplasts, decrease in chlorophyll biosynthesis and photosynthetic efficiency. Three principal components were extracted by principal component analysis. Correlation and regression analysis showed that heat tolerance is positively correlated with the plant height growth rate, fourth internode growth rate, the content of chlorophyll b, photosynthetic rate, stomatal conductance, transpiration rate, tuber number, and tuber yield, and negatively correlated with the cell membrane injury level. The nine traits are accurate and representative indicators for evaluating potato tolerance to heat stress and could determine a relatively high mean forecast accuracy of 100.0% for the comprehensive evaluation value. Through cluster analysis and screening, cultivar FA, D73, and C132 had the highest heat comprehensive evaluation value, which could be further selected as heat-resistant varieties. This study provides insights into the different physiological mechanisms and accurate evaluation methods of potato cultivars under heat stress, which could be valuable for further research and breeding.

## 1. Introduction

Potato (*Solanum tuberosum* L.) is a widely consumed staple food worldwide, originating from the Andes Mountains of South America, where it thrived in the low-temperature regions. Most cultivated germplasms are sensitive to high temperatures—temperature is considered to be the most important uncontrollable factor affecting potato growth and yield [1]. For most commercial potato cultivars, yield is optimal in the average daytime temperature range of 14–22 °C, outside of which, the yield declines sharply [2].

Global warming has become the main feature of climate change, significantly impacting agriculture, ecology, and threatening national and global food security by affecting agricultural production and related industries. The impact of global warming on potato production is expected to reduce global yield by 18–32% by 2050 [3]. Russet Burbank, a high yielding variety in the US and Canada, performs poorly under high temperatures, which could be observed from the reduced production acreage in Ontario during the hot summer of 2016 [4]. More than 60% of potatoes in China are grown in arid and semi-arid areas, and temperature and drought co-stress caused greater losses [5].

The effects of high temperatures on potato growth and development are numerous, high temperatures can stimulate stem growth, increase branching, inhibit root growth, reduce total leaf area, and increase the number of flowers per branch. Chloroplasts are damaged under high temperatures, resulting in a significant reduction in total chlorophyll content. This leads to a decrease in the photosynthetic rate and in the formation of assimilates [6], and causes a shift in assimilate distribution from tubers to leaves [7]. Heat stress results in a decrease in tuber induction and formation, as well as tuber growth and expansion. It also results in tuber malformation, necrosis, and dry matter content reducing [1]. Genetic diversity is the key to unravelling the morphological, physiological, biochemical, and genetic, as well as molecular traits in plants, thereby enabling the adoption of suitable crop improvement programs for refining the positive traits of existing crop plants, and also for breeding climate-smart crops under futuristic climatic conditions [8]. The sensitivity to heat stress varies with the plant and developmental stage, but its effects are highly dependent on genotype and species, exhibiting abundant interspecific and intraspecific variation [9]. Numerous differentially expressed genes involved in many biological processes and molecular functions, as well as differential metabolite accumulation, have been identified in response to mild to moderate heat stress in potato leaves and tubers [10]. Heat tolerance is a polygenic trait, so potato cultivars may exhibit a wide range of variations in response to heat stress. However, most studies of potato heat-stress responses have focused on limited germplasm data. The development of cultivars with tolerance is an effective way to mitigate the adverse effects of heat stress [11]. The selection and breeding of heat-tolerant potato cultivars are urgently needed to stabilize potato yields under current and future warm environments [4].

It is possible to select heat-tolerant cultivars based on the different responses of potato genotypes to temperature and using appropriate screening techniques. The yield of tubers is often regarded as the most convincing indicator to evaluate heat tolerance. Tang [4] evaluated the heat stress resistance of potato cultivars and identified cultivars that were relatively heat-tolerant or heat-sensitive. They found significant genotypic differences in the response of potato cultivars to heat stress in terms of chlorophyll content, plant height, and maximum tuber yield. The ability to form tubers under high temperatures can be used to screen heat-resistant potato cultivars [12,13]. In vitro culture systems employing continuous darkness and high sucrose concentrations in the culture medium or the addition of plant growth regulators allow for the screening of heat-tolerant potato cultivars based on tuberization indicators [14,15], and the in vitro-grown tubers were called microtubers [16]. In addition, photosynthesis is the prerequisite for crop growth and yield, and it is also one of the most sensitive physiological processes responsive to high temperature stress, physiological parameters related to photosynthesis can serve as an important indicators for evaluating crop heat tolerance [17]. The cell membrane is considered to be the main part of plants that is affected by heat stress. Heat stress can damage the integrity of the cell membrane structure, resulting in the loss of selective absorption and the breakdown of membrane permeability [18]. The changes in the cell membrane will affect other physiological activities of plants, such as reducing photosynthesis and mitochondrial activity [19]. Therefore, the electrical conductivity is often used to test the degree of damage to the cell membrane [20]. Within a certain temperature range, as the temperature increases, the stomatal conductance of plant leaves will increase, and the evaporation capacity will increase, thus accelerating the absorption of water, minerals, and gas exchange by plants, and also playing a role in cooling and preventing leaves from being burned by high temperatures. However, when the temperature reaches the stress temperature, the stomatal opening will decrease, the transpiration capacity will decrease, the leaf temperature will rise, and the normal physiological metabolic activities of plants will be disrupted [21].

In this study, a total of 93 cultivars were firstly screened using an in vitro tuber-inducing system depend on tuber formation, and the screened cultivars were further planted and exposed to heat stress, tuber yield and number, photosynthetic parameters, chlorophyll content, the cell membrane injury level of leaves were examined and determine whether significant variations exist in tested traits under heat stress conditions, the inter-correlation among these traits, and the identification of heat-tolerant cultivars, the evaluation method of potato cultivars were established. Our working hypothesis is that there is significant genotypic variation among potato cultivars in their response to heat stress in chlorophyll content, plant growth, tuber formation, and other traits, and relatively heat-tolerant cultivars could be identified based on these traits.

## 2. Results

### 2.1. Heat Stress Affects the Microtuber Development

**Figure 1:**
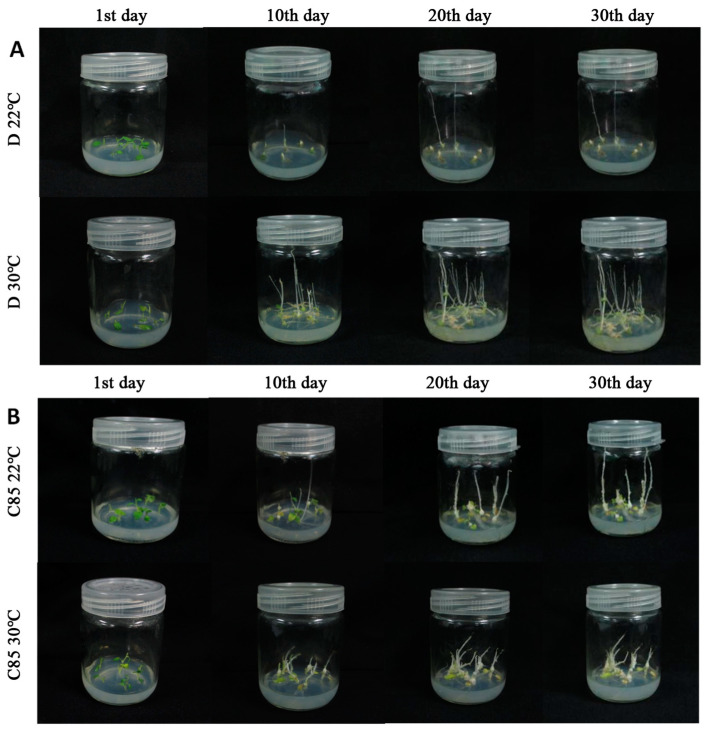
In vitro tuberization of two potato cultivars, D (**A**) and C85 (**B**) during 30 days of exposure to temperatures of 22 °C and 30 °C.

**Figure 2:**
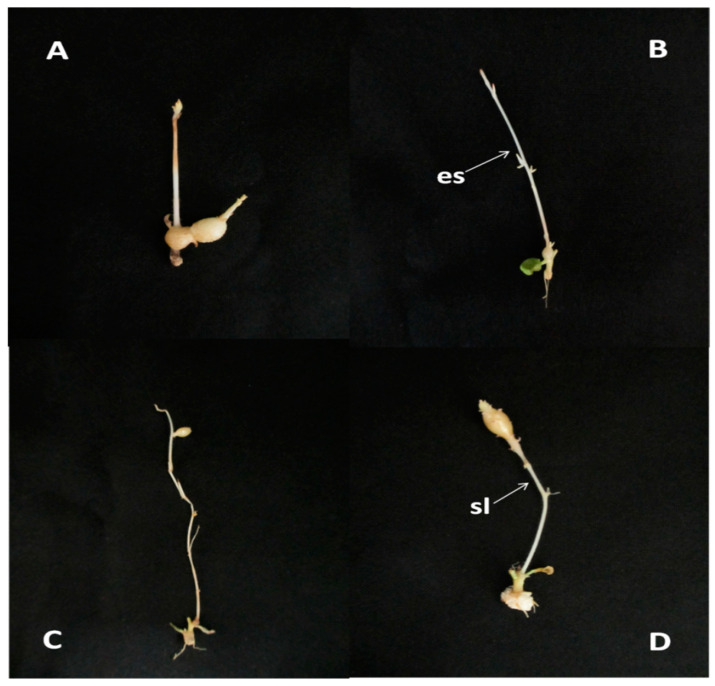
The in vitro microtuber types produced at 22 and 30°C. (**A**,**B**), basal microtuber; (**C**), medial microtuber; (**D**), apical microtuber; es, etiolated shoot from basal microtuber; sl, stolon-like shoot.

The microtuber development and the growth of shoots that mainly developed from an explant axillary bud (probably in some cases from microtuber apical bud) of 93 potato cultivars were investigated in this experiment (Table S1). Shoot development was observed earlier at 30 °C than those at 22 °C, explant developed more branches in the heat stress treatment compared with the control (Figure 1). Three types of in vitro microtubers were developed (Figure 2). The tubers formed from the axillary buds at the base of the stem were called basal microtubers (Figure 2A,B), and buds arise on the basal microtubers (Figure 2B). The microtubers formed from the axillary buds at the middle position of the stem were called medial microtubers (Figure 2C), and the microtubers formed from the axillary buds at the top position of the stem were called apical microtubers (Figure 2D). Explants of 93 potato cultivars mainly developed basal microtubers under heat stress (30 °C) and normal (22 °C) conditions for 30 days. In addition, cultivar D43 and D68 developed medial microtubers (Figure 2C), and cultivar D73, D79, D149, and FA developed apical microtubers (Figure 2D) under high temperature conditions, and the majority of the microtubers were non-sessile tubers. The number of basal microtubers accounts for the largest proportion of total microtubers, followed by medial microtubers, only few apical microtubers were developed among all cultivars.

In order to screen the most heat-tolerant potato cultivars, the effects of heat stress on tuberization were investigated, the percentage of explants with tubers under non-stress conditions (67.66%) was significantly greater than under heat stress conditions (27.98%). About 42 out of 93 potato cultivars developed microtubers (Table S2). The tuber number and tuber yield were significantly greater in the control conditions (0.73 ± 0.27 g per jar, 5.40 ± 2.53 tubers per jar) than in the heat stress conditions (0.48 ± 0.22 g per jar, 2.77 ± 1.76 tubers per jar), increasing by 48.61% and 34.23%, respectively (Table 1). Except for cultivar D72 for the tuber yield and D13 for the tuber number, the yield and number of microtubers decreased in all test cultivars after heat stress. The results revealed that D162 developed the most number (11 ± 0 per jar) and highest yield (1.86 ± 0.07 g per jar) under control conditions. The C56 and C122 developed the most number tubers at high temperature. The rates of tuber formation for explants of 15 potato cultivars was 100% during 30 days of cultivation under control temperatures, while only two cultivars including C56 and C122 had 100% rates of tuber formation under high temperature on day 30, respectively (Table S2).

### 2.2. The Developmental and Physiological Process Changed Significantly under Heat Stress Conditions

In this study, 42 out of 93 potato cultivars with tuberization were further selected to evaluate heat stress tolerance by simulating natural heat temperatures under the climate chamber. Two of the cultivars “Shepody” and “Atlantic” were excluded since plants were less healthy. The statistical analysis of the plant height growth rate, fourth internode growth rate, biomass of aerial parts, membrane damage, the content of chlorophyll, photosynthetic parameters, tuber yield, and number of 40 varieties was shown in Table 1. Heat tolerant coefficient (HTC) was calculated by 14 indicators (Table S3). The HTC value was varied among cultivars for each indicator. The average HTC in plant height growth rate, aerial parts fresh weight, aerial parts dry weight, chlorophyll b, photosynthetic rate (Pn), Gs, Tr, and fourth internode growth rate greater than 1.00, while chlorophyll a, total chlorophyll were less than 1.00. The coefficient of variation (CV) in all indices ranged from 14.64% to 63.47%. The CV of HTC value ranged from 1.4% to 142.05%. The fourth internode growth rate had the highest HTC value. The indicator of transpiration rate (Tr) and stomatal conductance (Gs) had the highest CV. After heat stress treatment, the plant height growth rate and fourth internode growth rate increased significantly (Table 1), the content of total chlorophyll and chlorophyll a decreased significantly. Heat stress also leads to increased aerial parts biomass and levels of cell membrane injury, and decreased photosynthetic parameters, tuber yield and number when compared to the control group (Table 1). However, the indicators in all varieties were not following the same trend; for example, 2 out of 40 varieties in the plant height growth rate were drastically decreased under heat stress compared with control.

### 2.3. Correlation Analysis between All Measured Trait Indicators

**Figure 3:**
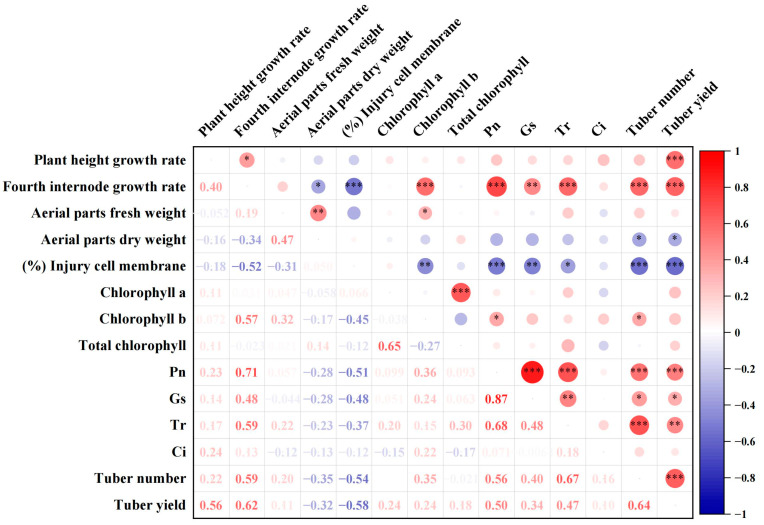
Correlation analysis of HTC between all the traits indices. *, *p* < 0.05; **, 0.001 < *p* ≤ 0.01; ***, *p* ≤ 0.001.

The Pearson correlation coefficient among all measured indicators were presented in Figure 3, the plant height growth rate was positively and significantly correlated with fourth internode growth rate and tuber yield; the content of chlorophyll b, photosynthetic rate (Pn) and stomatal conductance (Gs), transpiration rate (Tr), negatively and significantly correlated with the cell membrane injury level. The tuber yield was positively and significantly correlated with the plant height growth rate, fourth internode growth rate, Pn and Gs, Tr, and tuber number, negatively and significantly correlated with aerial parts dry weight and the cell membrane injury level. Intercellular carbon dioxide concentration (Ci) showed no significant correlation with any of the other indicators measured.

**Figure 4:**
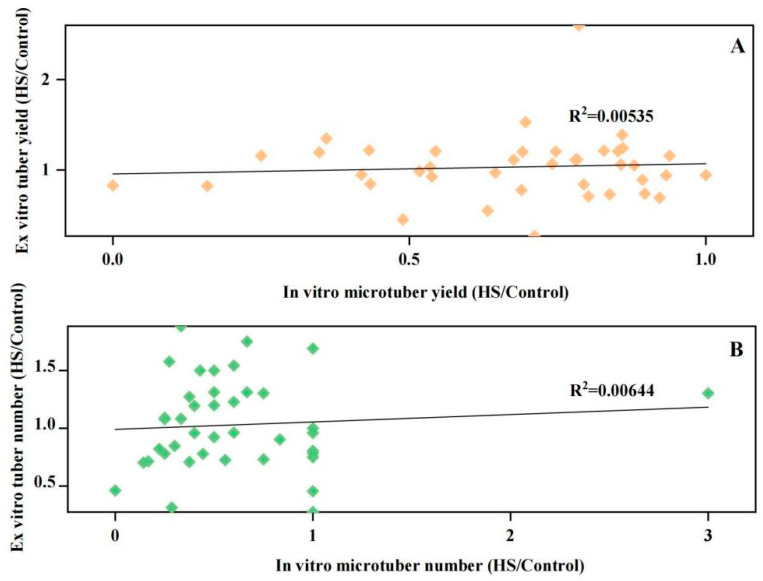
Correlation analysis of HTC represented by in vitro and ex vitro tuberization. (**A**), Correlation between the tuber yield; (**B**), Correlation between the tuber number.

Interestingly, the HTC of the tuber yield and number per plant under in vitro conditions did not show any significant correlation with that under ex vitro conditions (Figure 4), indicating the in vitro microtuberization system for potato heat stress tolerance screening needs to be verified by follow-up studies in greenhouses and field trials. Therefore, Pn and Gs, Tr, plant height growth rate, fourth internode growth rate, chlorophyll b, tuber number, aerial parts dry weight, and levels of cell membrane injury which related to the tuber yield under heat stress were used to evaluate the tolerance of potato cultivars by principal component analysis and multivariate statistical analysis.

### 2.4. Three Principal Components Analysis

Through principal component analysis, three top principal components were extracted based on the eigenvalue greater than 1.0, marked as F1 to F3, and the cumulative contribution rate of the top three main factors reached 76.46%, which together represented the information extraction of the 9 indicators under heat stress (Table 2). Under heat stress, the contribution rate of the first principal component was 52.22%, the F1 was mainly related to Pn, Tr, Gs, thus F1 was mainly related to photosynthetic parameter, so the first principal component was called the photosynthetic factor. F2 was mainly related to chlorophyll b and levels of cell membrane injury, which reflected the extent of damage to the chloroplasts and cell membranes, so it could be called the extent of cell damage indicator factor. F3 was mainly related to plant height growth rate, fourth internode growth rate, tuber yield and tuber number, which could be defined as a comprehensive morphological factor.

### 2.5. Heat Comprehensive Evaluation Value (HCEV), and Comprehensive Principal Components (F) Value Represented Higher Heat Tolerance

Since a single physiological index could not serve as a reliable indicator of heat tolerance in crops, comprehensive heat tolerance coefficient (CHTC), heat comprehensive evaluation value (HCEV), and comprehensive principal components value (F) based on multiple indexes were used for evaluating heat tolerance (Table 3). CHTC is the mean of comprehensive heat tolerance coefficient. HCEV and F were calculated based on the plant height growth rate, fourth internode growth rate, the content of chlorophyll b, Pn and Gs, levels of cell membrane injury, Tr, aerial parts dry weight, tuber number, and yield. The correlation analysis showed that the CHTC, HCEV, and F value were significantly and positively correlated with 8 indicators including the plant height growth rate, fourth internode growth rate, the content of chlorophyll b, Pn and Gs, and significantly and negatively correlated with the cell membrane injury level. The HCEV was significantly positively correlated with the CHTC (R^2^ = 0.91, *p* < 0.001) and F value (R^2^ = 1.00, *p* < 0.001) (Table 4). The CHTC of 40 potato germplasm resources ranged between 0.41 to 2.17. The HCEV of the 40 potato germplasm resources ranged from 0.13 to 0.93. The F value of the 40 potato germplasm resources ranged from −1.93 to 4.58, it has the same order in heat tolerance as HCEV value. However, the rank of CHTC and HCEV, or CHTC and F were not the same order. Cultivar FA and D73 exhibited a significantly higher CHTC, HCEV, and F value, but C132 and C20 showed a higher HCEV and F value and a lower CHTC. Therefore, a higher HCEV value indicates better heat tolerance, as it corresponded to a higher value in all target traits. The ranking of HCEV and F value showed that the potato cultivar FA, D73, C132, D54, C72, and C20 showed strong heat tolerance and C15, D211, and D showed less heat tolerance.

### 2.6. Screening of Heat Tolerance Traits by Stepwise Regression Analysis

The appropriate indicators were selected by stepwise regression analysis to comprehensively assess the heat tolerance of potato cultivars. The nine trait indicators were set as the independent variables, and the HCEV and F were set as the dependent variables. The models (R^2^ = 1.00) for the HCEV and F value were established under heat stress (Table 5). All nine indicators had significant effects on the comprehensive HCEV and F value (*p* < 0.001) and were identified as the key trait indices of a comprehensive evaluation for heat tolerance of potato.

### 2.7. The Different Heat-Tolerant Cultivars in Potato Were Grouped by Cluster Analysis Based on Comprehensive HCEV and F Values

**Figure 5:**
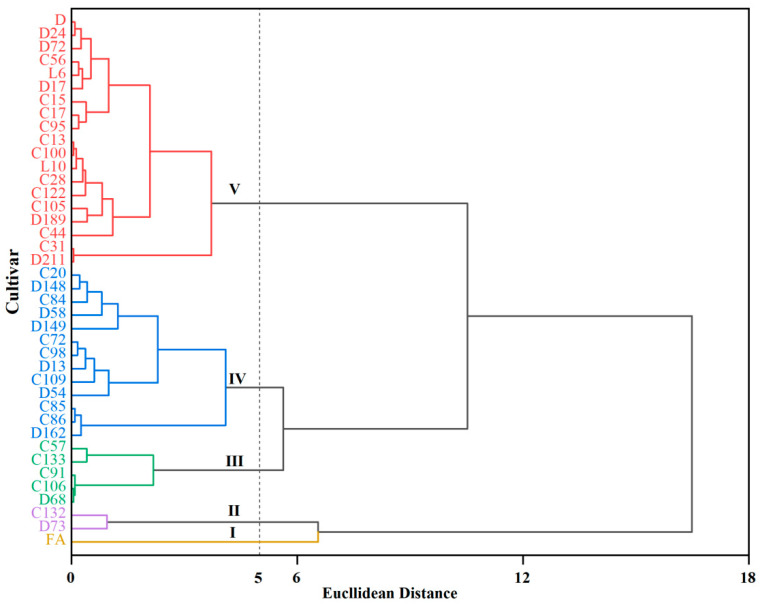
Cluster analysis for the heat tolerance of potato cultivars based on HCEV and F value.

Cluster analysis results of 40 potato cultivars based on the HCEV and F value were divided into five groups: high tolerance, tolerance, moderate tolerance, weak sensitivity, and sensitivity to heat (Figure 5). Group I, including one cultivar with the highest HCEV values and top F values, was confirmed to be highly tolerant to heat stress; where the FA had a F greater than 4.50, and had a HCEV greater than 0.80. Besides, the heat-tolerant cultivars in groups Ⅱ, Ⅲ, IV, and Ⅴ decreased in turn after group I. Furthermore, the D and C15 in group V had the lowest tolerance and highest sensitivity to heat stress, which had the lowest HCEV and F value.

## 3. Discussion

Considering the global climate warming, the probability of extreme high temperature weather continues to increase, and the duration is gradually prolonged. This has serious implications on the growth and development of crops, particularly in the north and northwest regions of China, where temperatures can reach 35 °C during potato growth periods. Heat stress can detrimentally affect crop growth and yield by altering physiological processes and metabolic pathways. These traits can be used to evaluate the heat tolerance of cultivars. Plant height, considered to be an indicator of sensitivity to heat stress [7]. The tuber yield and chlorophyll content are also considered key indicators of heat tolerance in potato cultivars [4]. Although researchers have developed different representative indicators from one or two aspects of morphology, physiology, and biochemistry to evaluate heat stress tolerance for potatoes, only partial indicators are applicable, and few unified and systematic indicators have been developed to evaluate heat stress tolerance. There is currently no evaluation index based on biological traits, physiological metabolism, or molecular markers. Evaluating heat tolerance based on a single index has certain limitations, the inclusion of too many indexes can increase workload and reduce the reliability of experiments. Therefore, it is particularly important to select reasonable indicators for comprehensive heat tolerance evaluation using multiple indexes.

In this study, 93 potato cultivars were studied under in vitro conditions, the tuber yield and number decreased significantly under heat stress, and 40 potato cultivars with tuber developed under in vitro conditions were selected to further study the heat tolerance of potatoes. The natural high temperature was simulated under an artificial climate chamber, we adopted a combination of morphological indicators (plant height growth rate, fourth internode growth rate, aerial parts dry weight, aerial parts fresh weight, tuber number, and tuber yield) and biochemical indices ((%)Injury cell membrane, Pn, Tr, Gs, chlorophyll a, chlorophyll b, total chlorophyll, and Ci) to evaluate heat tolerance, and demonstrated significant genotypic variation among potato cultivars for their response to heat stress.

Previous studies have shown that microtubers grown in vitro are essentially identical to tubers grown in the wild in terms of tissue structure, growth and development, and genetic stability [22]. In vitro screening for tolerance to heat stress are faster and reliable [23]. Khan et al. [13] suggested that inducing in vitro potatoes under high temperatures could be used as a method for screening heat-resistant cultivars. Pantelić et al. [16] assessed the potato heat tolerance based on tuberization, Désirée was designated as the heat-sensitive cultivar and Festival as the heat-tolerant cultivar. In recent years, frequent high-temperature weather events have been above 30 °C, when the night temperature exceeds 28 °C, the potatoes will not form tubers [24], and the current study used 30 °C as the threshold for screening and identifying potato heat tolerance. In this experiment, the microtuber number and weight decreased under heat stress incubation at 30 °C than those under control conditions. About 51 potato germplasm resources, including C5, C10, and C29, were unable to produce tubers under high temperature. However, 42 potato germplasm resources, including C13, C15, and C17, can produce tubers under high temperature. Comparisons of microtubers developed at 30 °C as heat treatment, with 22 °C as control condition, allowed to identify 42 heat tolerant and 51 susceptible potato cultivars. The results indicate that a temperature of 30 °C affected tuberization and allowed distinguishing heat stress responses among the cultivars. Three types of microtubers formed under in vitro conditions were also present in Pantelic et al. [16]. In these cultivars, the majority of tubers formed were sessile. This was in accordance with findings of Vreugdenhil et al. [25] and Xu et al. [14] on cv. Bintje, where uniform and synchronized formation of sessile tubers or tubers on short stolon-like structures was achieved. The suitable temperature for stolon growth of potatoes is about 5 °C higher than that of tuber expansion [26], so the high temperature may be more conducive to stolon growth and elongation. Only under heat stress, D73, D79, D149, and FA can form non-sessile microtubers. High temperatures lead to pre-mature sprouting in potatoes [1], and the length of the dormancy period is inversely proportional to the temperature [27]. The temperature optimum for breaking dormancy is 28 °C, and is 20–25 °C for sprout growth in potatoes [26]. Since our experimental temperatures were close to or within the range of the optimal temperatures for these processes, it could be hypothesized that these temperature treatments interfered with the onset of tuber dormancy and promoted the growth of tuber apical buds soon after tuber formation, eventually leading to the appearance of etiolated shoots on the basal microtubers.

Although the in vitro microtuberization system seems to be an efficient method to evaluate potato heat stress tolerance, this method has not yet been validated in ex vitro conditions. The traits of tuber number and yield under test-tube conditions cannot fully reflect the heat tolerance level of potato germplasm, and more key indicators are needed for further identification. The heat stress tolerance of these 42 potato cultivars were further evaluated. We employed 14 different parameters in terms of heat tolerant coefficient to evaluate potato tolerance to heat stress in ex vitro. High temperatures clearly reduced the tuber yield and number. However, we found the tuber yield and number per plant under in vitro conditions did not show any significant correlation with that under chambers conditions. Under heat stress, the number of tubers is significantly reduced, even leading to non-tuberization [28], while the tuber yield of potato cultivars with higher plant height is often not significantly reduced [4].

Photosynthesis is considered to be one of the most heat-sensitive physiological processes of plants. The photosystem II (PSII) complex, located on the thylakoid membrane, is highly sensitive to temperature during photosynthesis [29]. Heat stress can induce the conversion of active centers of PSII to inactive centers, leading to chlorophyll degradation. This results in a decrease in CO_2_ solubility, a reduction in the affinity of Rubisco for CO_2_, and a decrease in the thermal stability of key components in the photosynthetic system [30]. All of these factors can affect the photosynthetic rate of plants. Under high temperature conditions, plants with a strong heat tolerance can maintain a relatively non-stress physiological state, and maintain a high photosynthetic rate for guarantee the needs of plant growth or survival [31], high temperature stress may lead to partial stomatal closure, but plants with a strong heat tolerance can maintain relatively high transpiration rates and strong life activities, thus demonstrating strong adaptability to high temperatures [32]. When plants are subjected to high temperature stress, transpiration increases with an increasing temperature or prolonged stress time. This is because evaporating water can carry away some heat, thereby preventing high temperature from harming the plants [33]. In our study, the transpiration rate of some potato germplasm materials increased significantly after exposure to high temperature. High temperatures can cause the stomata to close, limiting the source of CO_2_ in leaves and hindering the smooth progress of photosynthesis. Our study showed that heat tolerance has significantly positively related the photosynthetic rate, stomatal conductivity, transpiration rate. Stomatal conductance, photosynthetic rate, and transpiration rate are positively correlated. The photosynthetic rate, stomatal conductance, transpiration rate, and intercellular CO_2_ concentration of heat-resistant potato germplasms C86, C132, FA, and D73 all increased, while the photosynthetic rate, stomatal conductance, transpiration rate, and intercellular CO_2_ concentration of heat-sensitive potato germplasm D211, C31, and Longshu No. 6 all significantly decreased.

As an important component of the photosynthetic system, chlorophyll is closely related to the photosynthetic capacity of plants. In this study, the decrease in chlorophyll index occurred in most cultivars under heat stress significantly. Under heat stress, a decrease in chlorophyll content is less pronounced in heat-resistant cultivars than in heat-sensitive ones [34]. In a computer-controlled greenhouse without drought stress, under thermal stress of 35 °C/28 °C (day/night), the average chlorophyll content index of highly tolerant potato cultivars was higher than that of the control [4]. The contents of chlorophyll a, chlorophyll b and total chlorophyll in heat-tolerant potato germplasm C132, FA and D73 in group I and Ⅱ were increased under high temperature stress; the contents of chlorophyll a, chlorophyll b and total chlorophyll in D211, C31 and L6 in group Ⅴ decreased significantly. The photosynthetic efficiency, stomatal conductance, and intercellular CO_2_ concentration also show varying degrees of reduction in some germplasm materials, ultimately resulting in decreased tuberization capacity and tuber yield [33]. Under high temperature conditions, both chlorophyll a and chlorophyll b may undergo destruction and degradation. The contents of chlorophyll a and chlorophyll b of some plants which have high heat tolerance remain relatively stable under high temperature conditions. These plants may possess efficient antioxidant systems and thermal adaptation mechanisms to mitigate the damage caused by high temperatures to chlorophyll. In this study, the content of chlorophyll b was used as one of the screening indicators for heat tolerance, the higher the contents of chlorophyll b, the more heat resistant the plant is.

When plants are subjected to stress, because the membrane structure is destroyed, the cell membrane permeability increases, the water-soluble substances in cells will have different extents of extravasation, leading to an increase in the relative electrical conductivity. The relative conductivity is an important indicator to measure the extent of cell damage [35]. Research by Yeh-Jin Ahn et al. [36] demonstrated that the heat-resistant cultivar Norchip exhibits stronger heat tolerance and lower levels of cell membrane injury compared to Désirée. Norchip demonstrates a stronger membrane stability under high-temperature stress. Our findings indicate a significant negative correlation between the strength of heat tolerance and the cell membrane injury level. The conductivity of low-heat resistant cultivars D211, C56, and C31 increased, indicating membrane damage.

High temperatures can cause plants to grow taller [37], and this was observed in heat-resistant rice cultivars under natural high temperature conditions [38]. Similar trends were observed in potato plants, which may be attributed to chlorophyll content and increased Pn [4]. In this study, the fourth internode growth rate of all tested potato germplasm increased under heat stress and significantly positively correlated with chlorophyll b and Pn. Potato germplasm with high heat tolerance like C132, FA and D73 showed high growth rate, while those with poor heat tolerance, like D211 and C56, showed a decreased plant height growth rate.

Furthermore, principal component analysis can simplify the original multiple variables into a few representative indices, namely principal components that are independent of each other [39]. Principal component analysis can effectively screen out the important and relevant traits for tolerance. Moreover, stepwise regression analysis can highlight the relevant indices and their specific correlations. Finally, the potato cultivars are objectively classified through cluster analysis. To comprehensively and systematically evaluate and screen the heat tolerance of potato cultivars, a multivariate analysis method was adopted in this study, including HCEV, F, correlation analysis, stepwise regression analysis, and cluster analysis. Principal component analysis was conducted based on 9 traits related to tuber number and yield, it was confirmed that the photosynthetic, degree of cell damage, and tuberization and plant-type factors all work together to improve potato tolerance for heat stress. Furthermore, correlation results supported this conclusion, as the HCEV and F value were significantly and positively correlated with plant height growth rate, fourth internode growth rate, chlorophyll b content, photosynthetic rate, stomatal conductance, transpiration rate, tuber number, and tuber yield, and negatively correlated with levels of cell membrane injury, which were identified as the important and relevant indicators for the comprehensive. All cultivars were successfully clustered into five groups based on the HCEV and F value. Among the high-resistance cultivars, C132, FA, and D73 had the highest HCEV and F value. Cultivar D and C15 were found to be the most sensitive cultivars to heat stress. The stepwise regression analysis showed that the 9 indicators can be used as reference indicators for heat tolerance evaluation of potato germplasm resources. The heat tolerance of 40 potato materials by the prediction equation was consistent with the HCEV and F value, indicating the high accuracy of the prediction equation.

It is important to note that this paper did not investigate the early maturity of each potato cultivar in the selection of experimental materials. Previous studies have suggested that early maturing potato cultivars exhibit a higher tolerance to high temperature stress, resulting in a higher tuber yield compared to late ripening cultivars [40,41]. This advantage can be attributed to the fact that early maturing cultivars reach the mature stage before the onset of high temperatures in summer, partially avoiding the adverse effects of heat stress [1]. In this study, the early maturing cultivar FA exhibited the highest heat tolerance, while the late maturing cultivars D, L6, and L10 showed high sensitivity to heat stress. However, Levy [1] found that the cultivars with the same maturity did not necessarily have the same response to high temperature stress. Zhang [42] found that the early maturity of cultivars and heat tolerance is not significantly related. The maturity periods, whether early, medium, or late, tend to vary randomly under high temperatures. Therefore, the heat tolerance identification method developed in this paper is likely to be applicable to potato cultivars across different maturity periods.

In addition, potatoes grown in hot summer often suffer from defects such as pre-harvest sprouting [42]. However, this study primarily focuses on the growth, physiological characteristics, and tuber traits of potato plants in relation to heat stress, and it does not investigate the correlation between heat stress and pre-harvest sprouting of potato tubers.

## 4. Materials and Methods

### 4.1. Plant Material and Heat Stress Application

A total of 93 potato cultivars including foreign elite lines, local landraces, and cultivars were clonally propagated in tissue culture on MS medium [43], containing 2% (*w*/*v*) sucrose under conditions of 16 h light and 8 h darkness at 22 °C. After four weeks, single-node stem cuttings (SNC, 15–20 mm in length) with one leaf were propagated on the tuberization medium in glass jars (six SNCs per jar, 30 mL of 8% MS medium) with vented polypropylene caps. The explants were grown in continuous darkness and exposed to temperature treatments of 22 ± 1 °C (control) or 30 ± 1 °C (heat stress treatment) in growth chambers. Two replicates were set up for both the control and treatment groups.

In total, 42 out of 93 preliminary screened potato germplasm resources were further clonally propagated at 2% MS medium under conditions of 16 h light and 8 h darkness at 22 °C for four weeks. Plants were transferred to 8 cm square pots containing vermiculite in a plant growth chamber and grown under 16 h light (10,000 Lux illumination) and 8 h darkness at 22 °C for four additional weeks. Plants were watered weekly. Subsequently, plants with a same growth status were transferred to circle pots (diameter: 24 cm) containing mix (vermiculite and substrate = 3:1) in growth chamber, and grown under 14 h photoperiod (10,000 Lux illumination) at the temperature of 25 °C/18 °C (day/night). After 3 weeks, plants were subjected to different temperature conditions for 60 days. Half of the plants were transferred to a new climate-controlled chamber and maintained at 35 °C/28 °C (day/night) for heat stress treatment. The left plants remained in the original chamber as a control treatment. Plants were fertilized daily with a half-strength solution of Holgland. After 60 days treatment, plants were maintained at control conditions for additional 60 days.

#### 4.1.1. Measurement of Microtuber Number and Yield

After 30 days of cultivation, the percentage of explants with microtubers, number of microtubers per jar, were determined. And the yield of tubers was measured using analytical balance (precision 0.0001 g). The final statistical results were based on the average value.

#### 4.1.2. Measurement of Growth Rate

The plant height on days 0 and 40, and the fourth internode growth rate on the 45th and 60th days were determined after heat stress treatment. The plant height growth rate and fourth internode growth rate were calculated as follows:

(1) $$Plant\;height\;growth\;rate=\frac{nth\;day\;height-mth\;day\;height}{n-m}$$

(2) Fourth internode growth rate=(nth day length−mth day length)/(n−m)

#### 4.1.3. Measurement of Chlorophyll Content

The fourth fully unfolded leaf of each plant from the top down was sampled after 60 days of heat treatment. The chlorophyll content was calculated according to the formula of Inskeep and Boom [44].

(3) Chlorophyll a=0.001×(13.95×OD664.5−6.88×OD647)/W

(4) Chlorophyll b=0.001×(24.96×OD647−7.32×OD664.5)/W

(5) Total Chlorophyll=Chlorophyll a+Chlorophyll b

#### 4.1.4. Measurement of Cell Membrane Injury

Cell membrane injury was determined using an electrical conductivity meter (DDS-307A, Yoke Instrument, Shanghai, China) according to previously described methods with some modifications [45]. The third leaf of each plant from the top down was sampled after 60 days of heat treatment, fresh leaf samples were washed three times with double-distilled water and blotted dry with clean filter paper. Then, eight leaf discs were sampled using a 6-mm-diameter punch from each plant, and put into tubes with 10 mL deionized water. The initial electrical conductivity (EC1) was measured after 12 h incubation at room temperature, the final electrical conductivity (EC2) was measured for all released electrolytes after the samples were autoclaved for 30 min at 100 °C. The extent of cell membrane injury was calculated as follows:

(6) $$(\%)\;Injury\;cell\;membrane=\frac{EC1}{EC2}\times 100\%$$

#### 4.1.5. Measurement of Photosynthetic Parameters

Photosynthesis parameters were determined using the Li-6400 portable photosynthetic meter (LI-COR Biosciences Inc., Nebraska, USA) after 60 days high temperature treatment, the potato plants to be tested were placed in light conditions for half an hour in advance for light adaptation, and the determination time was optimal from 8:30–11:30 a.m.

#### 4.1.6. Measurement of Biomass

The arial parts and roots of potato plants were sampled and cleaned separately after harvest. The fresh weight of roots and arial parts were measured separately using analytical balance (precision 0.0001 g). Dry weight was measured after drying the arial parts and root in an oven at 80 °C until a constant weight was achieved.

#### 4.1.7. Measurement of Tuber Number and Yield

The number of tubers formed on each plant was counted, and the yield of the tubers was measured using analytical balance (precision 0.0001 g).

### 4.2. Heat Tolerance Analysis

The HTC was the ratio of heat stress value (HS) to control value [46]. The calculation formula of the membership function value μ(X_ij_) and the heat comprehensive evaluation value (HCEV) are shown as follows [47]:

(7) $$CHTC=\frac{1}{n}\sum_{i=1}^{n}HTC$$

(8) $$\mu (x_{ij})=\left(x_{ij}-x_{min}\right)/{(x}_{max}-x_{min})$$

(9) $$V_{j}=\sqrt{\sum_{i=1}^{n}{\left(X_{ij}-\bar{X_{j}}\right)}^{2}/\bar{X_{j}}}$$

(10) $$W_{j}=V_{j}/\sum_{j=1}^{n}V_{j}$$

(11) $$F=\sum_{j=1}^{n}\left[x_{ij}\times W_{j}\right]$$

(12) $$HCEV=\sum_{j=1}^{n}\left[\mu (x_{j})\times W_{j}\right]$$

where X_ij_ was the value of ith cultivar in the jth indicator; X_min_, X_max_, V_j_ and $\bar{X_{j}}$ indicated the minimum value, maximum value, standard deviation coefficient, and average value for the jth indicator of all the cultivars, respectively, and W_j_ represented the yield of the jth indicator in all indicators. HTC and CHTC indicated the heat tolerance coefficient and comprehensive heat tolerance coefficient.

### 4.3. Data Analysis

The correlations among different measured and derived traits were estimated by calculating Pearson correlation coefficients using the statistical tool in Excel 2016. Data were subjected to analysis of variance (ANOVA) using SPSS software version 22.0 (IBM Corporation, Chicago, IL, USA), the significant difference between treatments was identified based on the LSD test at *p* ≤ 0.05. The software SPSS also was used to perform the principal component analysis and stepwise regression analysis. Meanwhile, OriginPro 2021 (OriginLab Inc., Northampton, MA, USA) was used to draw the figures.

## 5. Conclusions

Heat stress significantly causes the phenotypic and physiological changes in potatoes. The development of tubers under in vitro conditions could be an important indicator for the assessment of heat tolerance, used for initial screening and selection of 42 potato cultivars regarding heat tolerance. Under ex vitro conditions, the heat tolerance of 40 potato cultivars was analyzed using multivariate analysis methods. The results showed that potato cultivars with higher heat tolerance had higher plant height growth rate, fourth internode growth rate, chlorophyll b content, photosynthetic rate, stomatal conductance, transpiration rate, tuber number, and tuber yield, and lower levels of cell membrane injury. Upon exposure to high-temperature stress, the changes in plant height growth rate and fourth internode growth rate may offer potential information to identify heat tolerant potato germplasm. Correlation and regression analysis showed that the nine traits are accurate and representative indicators for comprehensive evaluating potato tolerance to heat stress. Overall, the potato germplasm C132, FA, and D73 were identified as the most heat resistant cultivars using the evaluation methods in this current study. The selection of these heat-resistant cultivars, together with the established method for identifying heat tolerance, could facilitate the selection of heat-tolerant parent cultivars for breeding programs. Moreover, it may assist in choosing suitable potato cultivars in regions with different degrees of heat stress and offer breeders information to identify the potentially heat tolerant germplasm.

## Figures and Tables

**Table 1:** Statistical analysis of main indexes in potato under heat stress.

Trait	Stress	Variation Range	Mean ± SD	CV (%)	*p* Value	HTC Mean	CV of HTC (%)
Microtuber number	Control	1–11	5.40 ± 2.53	46.85	-	0.61	79.80
	HS	0–8	2.77 ± 1.76	63.47			
Microtuber yield (g/per jar)	Control	0.27–1.86	0.73 ± 0.27	36.95	-	0.67	34.76
	HS	0–0.83	0.48 ± 0.22	45.93			
Plant height growth rate	Control	0.32–2.18	1.14 ± 0.48	41.43	**	1.57	55.89
	HS	0.67–2.76	1.53 ± 0.45	29.38			
Fourth internode growth rate	Control	0.14–0.86	0.30 ± 0.15	49.37	**	2.38	54.12
	HS	0.17–1.08	0.64 ± 0.29	44.68			
Aerial parts fresh weight (g)	Control	23.08–155.27	51.32 ± 21.01	40.42		1.19	33.32
	HS	1.75–9.36	55.64 ± 16.84	33.58			
Aerial parts dry weight (g)	Control	23.79–96.09	5.62 ± 1.91	29.88		1.10	36.55
	HS	2.27–12.45	5.74 ± 2.04	35.10			
(%) Injury cell membrane (%)	Control	10.74–21.84	14.84 ± 2.45	16.32		0.99	22.70
	HS	10.89–20.87	14.39 ± 2.27	15.61			
Chlorophyll a (mg/g)	Control	1.5–3.85	2.60 ± 0.50	18.86	*	0.95	18.94
	HS	1.6–3.56	2.42 ± 0.45	18.36			
Chlorophyll b (mg/g)	Control	0.08–1.16	0.68 ± 0.28	40.48		1.91	1.40
	HS	0.08–1.24	0.65 ± 0.29	43.22			
Total chlorophyll (mg/g)	Control	2.44–5	3.46 ± 0.51	14.64	**	0.89	21.45
	HS	2.14–4.54	3.04 ± 0.60	19.66			
Pn (µmol/m^2^/ s)	Control	3.21–21.55	11.49 ± 4.54	39.03		1.24	81.24
	HS	2.61–17.46	10.54 ± 4.23	39.60			
Gs (mol/m^2^/s)	Control	0.01–0.37	0.17 ± 0.10	60.56		1.94	142.05
	HS	0.02–0.44	0.16 ± 0.10	63.44			
Tr (mol/m^2^/s)	Control	0–0.01	0.002 ± 0.001	61.69		1.25	101.68
	HS	0–0.01	0.002 ± 0.001	63.22			
Ci (µmol/mol)	Control	84.78–312.72	254.89 ± 43.16	16.72		1.03	28.28
	HS	103.39–332.5	248.90 ± 52.56	20.85			
Tuber number	Control	2–20.33	9.62 ± 3.76	38.54		1.03	37.53
	HS	25.82–89.78	9.62 ± 4.90	25.01			
Tuber yield (g)	Control	1.67–27	56.04 ± 14.20	50.30		1.03	34.95
	HS	14.29–88.86	55.56 ± 15.56	27.66			

Pn, Photosynthetic rate; Gs, Stomatal conductance; Tr, Transpiration rate; Ci, Intercellular carbon dioxide concentration. Mean ± SD, mean (*n* = 3, biological replicates) ± standard deviation; *, *p* < 0.05; **, 0.001 < *p* ≤ 0.01. CV, coefficient of variation. HS, heat stress. HTC, heat tolerant coefficient.

**Table 2:** Loading matrix and the variance contribution rate of the principal component under heat stress.

	Principal Component			Weight of Principal Component		
Index	F1	F2	F3	F1	F2	F3
Pn	0.87	−0.31	0.21	0.40	−0.29	0.21
Fourth internode growth rate	0.86	0.07	−0.17	0.40	0.07	−0.17
Tuber number	0.79	0.04	0.02	0.36	0.04	0.02
Tuber yield	0.76	0.48	0.04	0.35	0.44	0.04
Tr	0.74	−0.15	0.38	0.34	−0.13	0.38
Gs	−0.73	0.04	0.29	−0.33	0.04	0.29
(%) Injury cell membrane	0.71	−0.43	0.27	0.33	−0.39	0.27
Plant height growth rate	0.417	0.78	0.13	0.19	0.72	0.13
Chlorophyll b	0.51	−0.15	−0.78	0.24	−0.14	−0.78
CR/%	52.22	13.03	11.21	-	-	-
CCR/%	52.22	65.24	76.46	-	-	-
Eigen value	4.84	1.18	1.04	-	-	-
Weight	0.69	0.17	0.14	-	-	-

F1, F2, F3, three top principal components; CR, contribution rate; CCR, cumulative contribution rate.

**Table 3:** The HCEV value, F value and CHTC of potato germplasm under heat stress.

Cultivar	HCEV		F		CHTC		Cultivar	HCEV		F		CHTC	
	Value	Rank	Value	Rank	Value	Rank		Value	Rank	Value	Rank	Value	Rank
D	0.17	39	−1.61	38	0.47	37	C109	0.42	16	0.49	17	0.91	17
C13	0.25	26	−0.94	26	0.57	26	C122	0.22	30	−1.24	31	0.57	27
C15	0.13	40	−1.96	40	0.51	34	C132	0.59	3	1.90	3	1.35	11
C17	0.20	33	−1.32	32	0.53	32	C133	0.46	12	0.89	14	1.20	12
C20	0.54	6	1.50	8	1.13	15	D13	0.38	17	0.51	16	1.41	10
C28	0.24	27	−1.00	27	0.58	24	D17	0.17	37	−1.52	36	0.44	39
C31	0.19	34	−1.50	35	0.50	35	D24	0.19	35	−1.43	34	0.45	38
C44	0.25	24	−0.94	25	0.56	29	D54	0.55	4	1.65	5	1.69	5
C56	0.21	31	−1.23	30	0.53	31	D58	0.49	11	1.12	12	1.14	14
C57	0.30	20	−0.46	19	0.56	28	D68	0.23	28	−1.05	28	0.49	36
C72	0.55	5	1.52	6	1.42	9	D72	0.30	21	−0.61	21	0.58	25
C84	0.45	15	1.06	13	1.66	7	D73	0.72	2	2.90	2	2.13	2
C85	0.51	9	1.51	7	1.77	4	D148	0.46	13	1.14	11	1.67	6
C86	0.52	8	1.33	10	1.18	13	D149	0.53	7	1.67	4	2.17	1
C91	0.25	23	−0.90	23	0.60	23	D162	0.50	10	1.41	9	1.57	8
C95	0.17	36	−1.69	39	0.55	30	D189	0.28	22	−0.71	22	0.82	18
C98	0.45	14	0.73	15	1.06	16	D211	0.17	38	−1.55	37	0.41	40
C100	0.25	25	−0.90	24	0.52	33	L10	0.21	32	−1.33	33	0.61	22
C105	0.22	29	−1.14	29	0.65	20	FA	0.93	1	4.58	1	1.90	3
C106	0.32	18	−0.36	18	0.65	21	L6	0.31	19	−0.48	20	0.66	19

HCEV, Heat comprehensive evaluation value; F, Comprehensive principal components value. CHTC, Comprehensive heat tolerant coefficient. The number in each column represents the score of principal component that was calculated based on mean (*n* = 3 biological replicates).

**Table 4:** Correlation matrix between comprehensive traits and measured index.

Trait	X_1_	X_2_	X_3_	X_4_	X_5_	X_6_	X_7_	X_8_	X_9_	HCEV	F
HCEV	0.52	0.87	−0.74	0.58	0.76	0.58	0.64	0.77	0.82	-	1
	***	***	***	***	***	***	***	***	***		***
F	0.5	0.87	−0.72	0.54	0.79	0.62	0.68	0.79	0.82	1	-
	***	***	***	***	***	***	***	***	***	***	
CHTC	0.37	0.84	−0.63	0.66	0.89	0.76	0.68	0.66	0.6	0.91	0.92
	*	***	***	***	***	***	***	***	***	***	***

X1, X2, X3, X4, X5, X6, X7, X8, X9 represent plant height growth rate, fourth internode growth rate, (%) injury cell membrane, chlorophyll b, Pn, Gs, Tr, tuber number and tuber yield, respectively. Same as below. *, *p* < 0.05; ***, *p* ≤ 0.001.

**Table 5:** The stepwise regression analysis for nine indicators with HCEV value or F value.

Models	R^2^	F	*p >* F
HCEV = 0.039 + 0.043X_1_ + 0.021X_2_ − 0.080X_3_ − 0.003X_4_ + 0.031X_5_ + 0.009X_6_ + 0.028X_7_ + 0.080X_8_ + 0.110X_9_	1.000	18,610.5	<0.001
F = −2.503 + 0.311X_1_ + 0.198X_2_ − 0.799X_3_ + 0.009X_4_ + 0.254X_5_ + 0.071X_6_ + 0.210X_7_ + 0.669X_8_ + 0.891X_9_	1.000	1,026,039.9	<0.001

## Data Availability

The data presented in this study are available upon request from corresponding author.

## Supplementary Materials

The following supporting information can be downloaded at: https://www.mdpi.com/article/10.3390/plants13010142/s1, Table S1: The potato cultivars were used in the experiment. Table S2: Effects of heat stress on potato tuberization parameters in vitro. Table S3: Heat tolerance coefficient (HTC) of each single index for 40 potato cultivars.
