# Supplementary material for: Multivariate Analysis Compares and Evaluates Heat Tolerance of Potato Germplasm
Source: Plants (Basel). 2024 Jan 4;13(1):142. doi: 10.3390/plants13010142 (PMC10781149; doi:10.3390/plants13010142)
Supplement: Supplementary file 1 [file plants-13-00142-s001.zip › plants-2709833-supplementary.pdf]

Supplementary Tables

Table S1. The potato cultivars were used in the experiment

Code	CIP code	Code	CIP code	Code	CIP code / Cultivar
C4	CIP392617.54	C105	CIP392032.2	D107	CIP395109.34
C5	CIP392634.52	C106	CIP392740.4	D117	CIP395448.1
C10	CIP393371.164	C109	CIP393613.2	D119	CIP396004.263
C13	CIP393280.64	C112	CIP397030.31	D120	CIP396004.337
C15	CIP391058.175	C114	CIP302428.20	D131	CIP396031.119
C17	CIP398192.213	C115	CIP302476.108	D147	CIP396241.4
C20	CIP398180.253	C116	CIP302499.30	D148	CIP396244.12
C28	CIP398208.58	C122	CIP304383.41	D149	CIP396268.1
C29	CIP398208.704	C123	CIP304383.80	D160	CIP397006.18
C30	CIP301024.14	C124	CIP304387.39	D162	CIP397039.53
C31	CIP301029.18	C129	CIP391930.1	D166	CIP398098.231
C35	CIP300054.29	C130	CIP391931.1	D167	CIP398098.570
C44	CIP385499.11	C132	CIP395438.1	D168	CIP398098.65
C50	CIP391382.18	C133	CIP394904.20	D188	CIP398208.670
C53	CIP392822.3	D13	CIP301045.74	D189	CIP399001.44
C56	CIP394034.65	D17	CIP303381.106	D197	CIP399062.118
C57	CIP394034.7	D24	CIP304351.31	D199	CIP399067.22
C63	CIP394614.117	D31	CIP304394.56	D203	CIP399075.32
C65	CIP395186.6	D43	CIP381403.16	D210	CIP399085.30
C70	CIP395432.51	D53	CIP391580.30	D211	CIP694474.16
C72	CIP395436.8	D54	CIP391585.179	L3	Longshu No.3
C84	CIP397065.2	D55	CIP391585.5	L6	Longshu No.6
C85	CIP397067.2	D58	CIP392285.72	L10	Longshu No.10
C86	CIP397069.5	D67	CIP393077.54	L12	Longshu No.12
C89	CIP397079.26	D68	CIP393079.24	GN3	Gannong No.3
C91	CIP397098.12	D72	CIP393220.54	GN6	Gannong No.6
C95	CIP397196.8	D73	CIP393242.50	Shepody	Shepody
C97	CIP398014.2	D79	CIP393349.68	Atlantic	Atlantic
C98	CIP388611.22	D87	CIP393536.13	FA	Favorita
C100	CIP389468.3	D88	CIP393617.1	QS9	Qingshu No. 9
C101	CIP390637.1	D96	CIP395011.2	D	Désirée

Table S2. Effects of heat stress on potato tuberization parameters in vitro

	Explants with tubers (%)		Tuber number per jar		Tuber yield per jar (g)	
Cultivar	Control	HS	Control	HS	Control	HS
Désirée	66.67%	0%	4±1.41	0±0	0.71±0.27	0±0
C13	8.33%	8.33%	1±1.41	1±1.41	0.33±0.47	0.31±0.44
C15	33.33%	16.67%	2±0	2±0	0.38±0.085	0.27±0.028
C17	100.00%	66.67%	9±1.41	4±0	1.01±0.042	0.81±0.03
C20	100.00%	16.67%	6±0	2±0	0.87±0.01	0.72±0.06
C28	66.67%	8.33%	4±0	1±1.41	0.53±0.03	0.23±0.33
C31	58.33%	8.33%	7±1.41	1±1.41	0.68±0.06	0.57±0.81
C44	91.67%	83.33%	9±1.41	5±1.41	0.76±0.04	0.49±0.17
C56	100.00%	100.00%	8±0	8±1.41	0.97±0.06	0.77±0.06
C57	100.00%	16.67%	9±1.41	2±0	0.76±0.01	0.61±0.01
C72	16.67%	8.33%	2±2.83	1±1.41	0.37±0.52	0.16±0.22
C84	100.00%	16.67%	6±0	2±0	0.68±0.06	0.37±0.01
C85	66.67%	33.33%	4±1.41	3±0	0.93±0.20	0.8±0.03
C86	16.67%	16.67%	2±0	2±0	0.49±0.03	0.42±0.06
C91	83.33%	16.67%	5±1.41	2±0	0.93±0	0.39±0.14
C95	58.33%	16.67%	7±1.41	2±0	0.45±0.06	0.22±0.04
C98	66.67%	16.67%	4±0	2±0	0.95±0.06	0.71±0.07
C100	66.67%	25.00%	4±0	3±0	0.8±0.10	0.43±0.04
C105	100.00%	8.33%	6±0	1±1.41	0.69±0.04	0.11±0.16
C106	100.00%	25.00%	10±0	3±0	0.85±0.04	0.63±0.03
C109	83.33%	25.00%	5±1.41	3±0	0.92±0.11	0.72±0.04
C122	100.00%	100.00%	8±1.41	8±1.41	0.75±0.08	0.7±0.01
C132	83.33%	25.00%	5±1.41	3±0	0.69±0.18	0.48±0.03
C133	66.67%	8.33%	4±0	1±1.41	0.72±0.04	0.18±0.25
D13	16.67%	16.67%	2±0	2±0	0.27±0.01	0.23±0.01
D17	100.00%	25.00%	6±0	3±0	0.87±0.07	0.55±0.07
D24	100.00%	25.00%	8±0	3±0	0.74±0.06	0.51±0.01
D54	66.67%	8.33%	4±0	1±1.41	0.69±0.03	0.24±0.34
D58	100.00%	25.00%	8±2.83	3±0	0.68±0.03	0.47±0.10
D68	16.67%	16.67%	2±0	2±0	0.41±0.01	0.32±0.01
D72	100.00%	83.33%	6±0	5±1.41	0.72±0	0.72±0.11
D73	100.00%	66.67%	8±2.83	4±0	0.68±0.03	0.46±0.01
D148	25.00%	16.67%	3±0	2±0	0.58±0.04	0.51±0.03
D149	83.33%	16.67%	5±1.41	2±0	0.86±0.08	0.46±0.03
D162	100.00%	25.00%	11±0	3±0	1.86±0.07	0.67±0.16

D189	83.33%	25.00%	5±1.41	3±0	0.75±0.11	0.67±0.06
D211	16.67%	16.67%	2±0	2±0	0.39±0.04	0.35±0
L10	41.67%	41.67%	5±0	5±0	0.9±0.11	0.83±0.10
FA	58.33%	25.00%	7±0	3±0	0.98±0.14	0.77±0.10
Shepody	16.67%	16.67%	3±0	2±0	0.58±0.07	0.3±0.01
L6	25.00%	16.67%	4±1.41	0±0	0.71±0.27	0±0
Atlantic	100.00%	25.00%	1±1.41	1±1.41	0.33±0.47	0.31±0.44
Average value	67.66%	27.98%	5.40±2.53	2.77±1.76	0.73±0.27	0.48±0.22

HS, heat stress

Table S3. Heat tolerance coefficient (HTC) of each single index for 40 potato cultivars

Cultivar	X_1	X_2	X_3	X_4	X_5	X_6	X_7	X_8	X_9	X_{10}	X_{11}	X_{12}	X_{13}	X_{14}
D	1.5	1.02	1.19	1.43	1.2	0.95	0.96	0.95	0.31	0.21	0.15	0.64	0.46	0.83
C13	0.67	1.16	1.44	1.2	1.17	0.78	0.79	0.78	0.58	0.73	0.76	0.77	1	1.16
C15	1.06	1.9	0.98	1.31	1.19	0.9	0.21	1.31	0.62	0.88	0.68	0.9	0.28	0.27
C17	0.91	1.23	1.09	1.5	1.08	0.74	0.76	0.74	0.8	0.6	0.55	1.26	0.78	0.71
C20	1.81	3.17	1.66	0.94	0.77	0.77	0.77	0.77	2.00	2.17	2.02	0.88	1.88	1.21
C28	1.61	1.15	0.57	0.49	1.04	0.98	1.1	1.04	0.66	0.37	0.6	0.87	0.78	0.84
C31	1.47	1.32	0.81	0.96	1.36	0.85	0.11	0.68	0.44	0.43	0.5	1.22	0.7	0.73
C44	1.16	1.05	1.09	0.71	1.00	1.07	0.14	0.85	0.72	1.13	0.88	1.02	0.73	0.97
C56	0.57	2.31	1.12	1.07	1.04	0.8	0.82	0.8	0.4	0.35	0.31	0.95	0.79	0.84
C57	0.94	1.16	1.37	2.01	0.72	1.14	0.87	1.14	0.56	0.9	0.43	0.91	0.82	1.39
C72	2.48	3.03	1.49	1.42	0.99	0.63	1.44	0.68	2.57	4.46	2.4	1.16	1.31	1.22
C84	1.01	4.47	1.18	1.12	0.81	0.97	8.56	0.76	2.76	1.92	1.43	1.19	1.08	1.21
C85	3.02	5.15	1.96	1.09	0.94	0.93	8.76	0.74	1.3	1.58	1.46	0.96	1.3	1.24
C86	1.65	2.33	1.54	1.19	0.64	1.07	1.25	1.12	1.31	3.11	3.48	1.23	1.69	1.06
C91	1.00	1.21	1.23	1.11	1.06	1.21	0.87	1.19	0.61	0.81	0.93	0.99	0.96	0.95
C95	2.67	1.14	1.18	1.55	1.46	1.13	0.16	0.92	0.87	0.44	0.26	0.44	0.31	0.45
C98	1.59	2.47	1.15	1.24	0.9	1	1.17	1.03	1.73	2.2	2.36	0.94	1.2	1.2
C100	1.24	2.39	0.93	1.25	0.96	1.01	0.13	0.81	0.55	0.19	0.18	0.38	0.73	0.93
C105	1.12	2.02	0.85	0.89	1.21	0.91	1.26	0.97	0.82	0.66	0.5	0.75	0.71	0.82
C106	2.65	1.37	1.79	1.72	0.9	0.85	0.84	0.85	0.45	0.53	0.41	1.07	0.85	1.07
C109	1.9	3.28	1.35	1.46	0.85	1.08	1.09	1.08	1.14	1.05	1.14	0.97	1.23	1.12
C122	1.09	1.18	1.23	0.79	1.46	1.4	0.13	0.76	0.62	0.8	0.79	1	0.96	0.94
C132	1.61	3.56	1.71	1.35	0.92	1.25	1.23	1.25	2.53	2.64	3.31	1.26	1.54	1.53
C133	1.28	3.93	1.16	1.12	0.97	0.7	1.5	0.69	2.34	2.33	2.2	1.39	1.09	1.16
D13	0.95	2.63	1.74	0.98	0.69	0.77	8.39	0.62	1.36	2.07	1.1	1.03	1.3	1.21
D17	0.95	1.18	1.6	1.99	1.01	0.8	0.8	0.8	0.31	0.1	0.28	0.55	0.92	0.55
D24	1.09	1.02	1.34	1.3	1	0.79	0.8	0.79	0.36	0.26	0.23	0.75	0.71	0.78
D54	1.47	3.9	1	0.66	0.7	0.66	1.01	0.65	2.88	10.37	1.04	0.59	1.08	1.19
D58	1.67	4.05	1.14	0.68	0.86	0.95	1.01	0.96	1.66	1.88	2.38	1.05	1.27	1.2
D68	1.03	1.1	1.6	1.02	1.01	1.15	0.83	1.16	0.39	0.32	0.32	0.91	0.8	1.11
D72	3.22	1.06	0.65	0.86	1.04	0.66	0.66	0.66	0.15	0.06	0.09	2.62	0.9	0.94
D73	1.08	3.56	1.21	0.82	0.93	1.13	1.03	1.17	4.09	10	6.47	1.05	1.5	1.11
D148	1.61	4.18	1.18	0.82	0.76	0.91	8.65	0.73	1.47	1.78	2.54	2.14	1.31	1.05
D149	1.63	2.94	0.85	0.84	0.65	1.1	7.23	0.89	3.83	11.74	0.08	1.1	1.19	1.03
D162	1.71	4.21	1.83	0.74	0.83	1	7.35	0.81	1.23	1.45	2.28	0.77	1.58	1.35
D189	1.66	2.89	0.8	0.59	1.52	1.03	0.94	0.75	1.01	0.9	0.77	1.15	0.96	0.89

D211	0.55	1.5	0.55	0.68	1.08	0.86	0.11	0.68	0.37	0.27	0.37	0.99	0.75	0.74
L10	1.76	1.69	1.29	2.02	1.19	1.04	0.83	0.99	0.54	0.71	0.62	1.4	0.46	0.69
FA	5.42	5.17	0.55	0.37	0.67	1.22	1.22	1.22	2.69	4.61	2.72	1.04	1.5	2.6
L6	1.18	1.21	0.15	0.8	1.26	0.82	0.86	0.83	0.54	0.63	0.86	0.85	1.75	0.99
Average value	1.57	2.38	1.19	1.1	1	0.95	1.92	0.89	1.24	1.94	1.25	1.03	1.03	1.03
CV	0.56	0.54	0.33	0.37	0.23	0.19	1.41	0.22	0.81	1.42	1.02	0.38	0.38	0.35

X_1, X_2, X_3, X_4, X_5, X_6, X_7, X_8, X_9, X_{10}, X_{11}, X_{12}, X_{13}, X_{14} represent plant height growth rate, fourth internode growth rate, aerial parts fresh weight, aerial parts dry weight, (%) injury cell membrane, chlorophyll a, chlorophyll b, total chlorophyll, Pn, Gs, Tr, Ci, tuber number and tuber yield, respectively.
